# Evaluation of possible toxic effects of spearmint (*Mentha spicata*) on the reproductive system, fertility and number of offspring in adult male rats

**Published:** 2014

**Authors:** Fatemeh Nozhat, Sanaz Alaee, Khodabakhsh Behzadi, Najmeh Azadi Chegini

**Affiliations:** 1 *Department of Biology, Payame Noor University (PNU), IRAN*; 2 *Department of Reproductive Biology, School of Advanced Medical Sciences and Technologies, Shiraz University of Medical Sciences, Shiraz, I. R. Iran*; 3 *Department of Biology, Sciences and Research Branch, Islamic Azad University, Tehran, I. R. Iran*; 4 *Biology Group, Department of Education, Fars, I. R. Iran*

**Keywords:** *Fertility*, *Male*, *Spearmint*, *Sperm*, *Toxicity*

## Abstract

**Objective**: In this study we investigated the effects of spearmint (*Mentha spicata *Labiatae) on the reproductive system, fertility and number of offspring in adult male rats.

**Materials and Methods:** Adult Wistar male rats in one control (C) and three experimental groups (I, II and III) received 0, 10, 20 and 40 mg/kg spearmint extract orally for 45 days, respectively. Following this treatment, the animals’ weights, and the standard weight of reproductive tissues, sperm count, sperm motility and serum testosterone concentration were measured, and reproductive tissues were examined histopathologically. To evaluate the effects of spearmint on fertility of male rats and growth of their offspring, male rats of the control and experimental groups mated with untreated female rats.

**Results:** Results showed that spearmint did not affect the rats’ body and reproductive tissue weights. The sperm count, fast and slow progressive motility of sperm and serum testosterone concentration decreased while number of non-progressive sperm and immotile sperm increased in the experimental groups compared to the control group, but none of these changes were statistically significant. Histopathological studies showed no severe changes in reproductive tissues between control and experimental groups. Number and growth of offspring born from mating of male rats with untreated female rats showed no difference.

**Conclusion:** We concluded that spearmint has no significant toxic effect on the reproductive system, fertility and number of offspring in adult male rats at the above mentioned dose levels. However high levels of this extract may have adverse effects on male fertility.

## Introduction

Since Iran has many diverse herbal plants, traditional medicine is widely used for treatment of many diseases in this country. The Lamiaceae family is one of the families of flowering plants (Naghibi et al., 2005 ▶) and genus Mentha, an important member of this family, has 6 species in the flora of Iran. These species have great importance in folk medicine and are available in traditional medicinal plant stores and local markets (Amin, 2005 ▶). Spearmint is one of these species, with a good flavor and fragrance, used worldwide in pharmaceutical preparations, confectionery and food industries, and also in hygiene and cosmetic products (Kumar et al., 2008 ▶; Spirling and Daniels, 2001 ▶). Some studies have been shown that spearmint oil has anti-fungal, anti-microbial, anti-inflammatory, anti-tumor and antioxidant activity (Guimaraes et al., 2011 ▶; Lixandru et al., 2010 ▶; Mazzio and Soliman, 2009 ▶; Pearson et al., 2012 ▶; Soković et al., 2009 ▶). Furthermore, various beneficial medicinal effects of spearmint have been found, such as preventing chemotherapy-induced nausea and vomiting (CINV), treatment of respiratory and digestive system disorders, hypertension, anxiety and even for relieving menstrual pain (Cakilcioglu et al., 2011 ▶; Gomez Estrada et al., 2011 ▶; Karousou et al., 2007 ▶; Rokaya et al., 2010 ▶; Tayarani-Najaran et al., 2013; Vejdani et al., 2006; Yoney et al., 2010 ▶). Spearmint is mainly recommended for its antispasmodic effects, which are related to its carvone content, the most important constituent of spearmint (29–74%). Spearmint also contains 4–24% limonene, 0.21–2.1% volatile oil and 3–18% cireole (Baser, 1993). 

There is some evidence which show that despi te its beneficial effects, spearmint has some toxic and adverse effects. Severe histopathological changes in kidney, liver and uterus tissue (Akdogan et al., 2004a ▶; Akdogan et al., 2003 ▶; Guney et al., 2006 ▶) and also contact allergic reaction to the leaves of spearmint have been reported (Bonamonte et al., 2001). Furthermore, daily consumption of four cups of spearmint tea can diminish libido in men (Akdogan et al., 2007 ▶). In one study, Akdogan showed that spearmint herbal tea has adverse effects on testicular tissue and testosterone level, and alters the level of follicular stimulating hormone (FSH) and luteinizing hormone (LH) (Akdogan et al., 2004b ▶; Kumar et al., 2008 ▶).

Since spearmint is widely used for digestive problems in Iran, in this study we investigated the possible toxic effects of this agent on the male reproductive system by evaluation of reproductive tissues’ histopathology, plasma testosterone concentration, sperm concentration, spermmotility and number of offspring in adult male rats.

## Materials and Methods

**Preparation of hydroalcoholic extract of spearmint**

Fresh spearmint was purchased from a local market source in Shiraz*. *The plant’s identity was confirmed by a botanist in the Biology Department, Payame Noor University, Shiraz, Iran, and a voucher specimen (1275) was deposited in this Department. Hydroalcoholic extract was prepared using the maceration method. Leaves were cleaned and dried under shade at room temperature. The dried leaves of the plant were powdered (400 g) and macerated in 1400 ml ethanol for 3 days. Then the solution from the total extract was filtered with filter paper, concentrated by evaporation and stored in refrigerator until used for our experiments. The yield (w/w) of the solution was 9.6% (g/g).

**Experimental design**

Adult Wistar male rats of proven fertility, 8–10 weeks old age and weighing 200–250 g were kept in laboratory conditions for adaptation two weeks before experiments. They were maintained in a well-ventilated animal house under standard conditions and controlled temperature (22–24 °C), with periods of 12 hours light and 12 hours darkness. The animals had sufficient access to food and water throughout the study. The weights of animals were recorded before and after the experiments. The animal experiments were performed according to the principles of the care and use of laboratory animals established by the National Institutes of Health (NIH Publication, 1985). Then male rats were randomly divided into one control (C) and three experimental groups (I, II, III).

There were 15 animals in each group (9 animals for histopathological and sperm parameters evaluation and 6 animals for mating with untreated female rats). Group Ι received 10 mg/kg, group II received 20 mg/kg and group Ш received 40 mg/kg of spearmint leaf extract orally for 45 days. The control group received 1 ml of distilled water daily.

**Hormone assay**

After 45 days, animals were sacrificed using diethyl ether. Blood samples were collected by dorsal aorta puncture, centrifuged at 3000 rpm for 15 min, and serum was separated. The concentration of serum testosterone was assayed by solid phase radioimmunoassay (RIA) method.

**Standard weight of reproductive tissues**

After scarifying the animals and collecting blood samples, testes, epididymis, seminal vesicles and prostate were removed and weighed. The standard weights of right testis, epididymis, seminal vesicle and prostate was calculated by following formula: [tissue weight (g) / body weight (g)] × 100.

**Histopathological studies**

The testes, epididymis, seminal vesicles and prostate of each rat were fixed in 10% buffer formalin solution. Tissues were processed for preparation of paraffin blocks, which then were sectioned at a thickness of 6 µm using a microtome, and stained with hematoxylin and eosin.

**Sperm movement and count**

During dissection of each rat, the distal portion of each vas deferens (1 cm) was removed and placed in a falcon tube contained 5 ml Hanks’ solution at 37 ºC for capture of sperm exiting from the vas deferens (Seed et al., 1996 ▶). After 3 minutes, one drop of Hanks’ solution containing sperm was placed on a microscope slide and for each animal, movement of 100 spermatozoa were assessed by light microscope at 40x magnification. Sperm movements were divided into 4 grades of fast progressive movement, slow progressive movement, non-progressive movement and immotile sperm, according to the WHO Laboratory Manual for the Examination and Processing of Human Semen (WHO, 2010 ▶). After 10 minutes, sperm count was assessed by putting one drop of sperm suspension on a hemocytometer (Da Silveira et al., 2003 ▶). Total sperm number was calculated using the following formula: A= B×C×D,

where

A: total sperm number in 1 cm of vas deferens

B: total sperm number in 0.1 mm^3^ of solution

C: deep factor

D: dilution factor= 5000mm^3^

**Animals’mating**

To evaluate the effect of spearmint on the fertility of male rats and the growth of their offspring, following the last day of spearmint administration, 6 male rats from each group were cohabitated with proestrus untreated females rats for mating, per the following design:

1) 6 male rats of group C mated with 6 untreated female rats 

2) 6 male rats of group I mated with 6 untreated female rats

3) 6 male rats of group II mated with 6 untreated female rats

4) 6 male rats of group III mated with 6 untreated female rats

Vaginal smear was examined every morning to determine positive mating. After completion of the duration of the female rats’ pregnancies, the number, weight and crown-rump length (CRL) of offspring from each pregnant female rat were recorded (Monsefi et al., 2010 ▶).

**Statistical analysis**

Statistical analysis was done using SPSS 11.5 software. For data analysis, we used one-way ANOVA followed by the Tukey post hoc test. Results were expressed as mean ± SEM (standard error of the mean) and p<0.05 was considered statistically significant.

## Results

Comparison of body weight of rats at the beginning and end of the experiment showed no significant difference between control and experimental groups. The standard weight of right testis, epididymis, seminal vesicle and prostate had no significant change in experimental groups when compared to the control group (Table 1). The serum testosterone concentration, sperm count and the number of sperm with fast and slow progressive movement were lower in experimental groups compared to the control group but not statistically significant (Table 2). The number of non-progressive and immotile sperm was higher in experimental groups compared to the control group, but none of these changes were statistically significant (Table 2).

**Table 1:** The standard weight (g) of right testis, seminal vesicle, epididymis and prostate in control, group I, group II and group III male rats. (n=9 for each group)

**Groups**	**Right testis standard weight**	**Right seminal vesicle standard weight**	**Right epididymis standard weight**	**Prostate standard weight**
Control	1.19±0.18	1.04±0.41	0.27±0.04	0.51±0.21
Group I	1.19±0.10	1.08±0.26	0.26±0.02	0.60±0.22
Group II	1.18±0.24	1.07±0.37	0.26±0.05	0.64±0.12
Group III	1.29±0.13	1.21±0.32	0.28±0.04	0.63±0.19

Values represent mean ± SEM. There was not statistical difference between the data of different groups.

**Table 2:** Serum testosterone concentration (ng/ml), sperm count and sperm motility in control, group I, group II and group III male rats. (n=9 for each group

**Groups**	**Serum testosterone concentration **	**Sperm count** **(×10** ^5^ **)**	**Sperm motility**			
			**Fast progressive movement (%)**	**Slow progressive movement (%)**	**Non-progressive movement (%)**	**Immotile sperm (%)**
Control	3.19±0.48	5.32±3.72	29.88±7.65	26.22±4.96	26.88±1.20	17.00±7.12
Group I	2.69±0.64	4.81±2.60	23.66±7.19	22.44±7.87	32.55±1.15	21.33±8.17
Group II	2.72±0.37	4.56±3.63	23.66±4.70	21.55±5.40	30.22±1.21	24.55±8.90
Group III	2.63±1.03	4.37±3.33	24.44±4.15	21.00±6.00	33.11±1.16	22.33±7.40

Values represent mean ± SEM. There was not statistical difference between the data of different groups.

**Figure 1:**
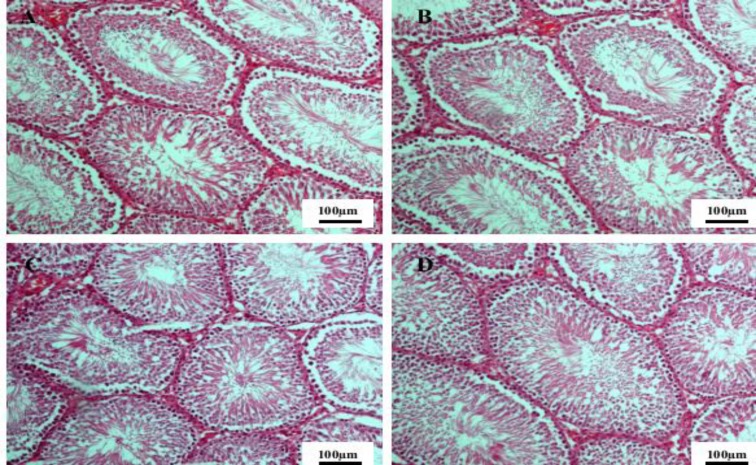
The seminiferous tubules in the control group (A) group I (B) group II (C) and group III (D) of male rats. Hematoxylin and eosin staining, 10x magnification

**Figure 2:**
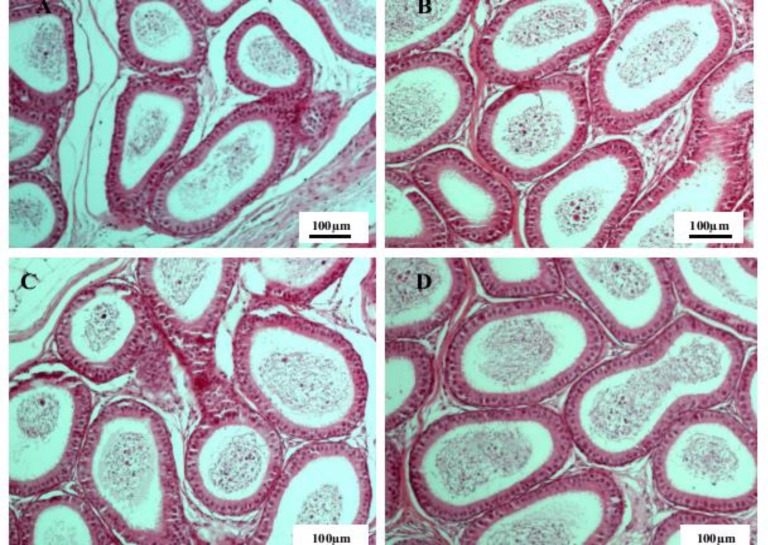
The epididymis tissue in the control group (A) group I (B) group II (C) and group III (D) of male rats. Hematoxylin and eosin staining, 10x magnification

Histopathological studies showed no change in the structure of reproductive tissues between experimental groups and the control group. All seminiferous tubules of testis tissue had normal histopathological features in control and experimental groups. Spermatogenic cells at all stages of spermatogenesis (spermatogonia, primary spermatocytes, secondary spermatocytes, spermatids and spermatozoids) were seen in these tubules, demonstrating that the normal spermatogenesis process has occurred in these animals (Figure 1). The epididymal tissue in all groups showed normal structure with pseudostratified columnar epithelium and normal sperm density (Figure 2). 

**Figure 3:**
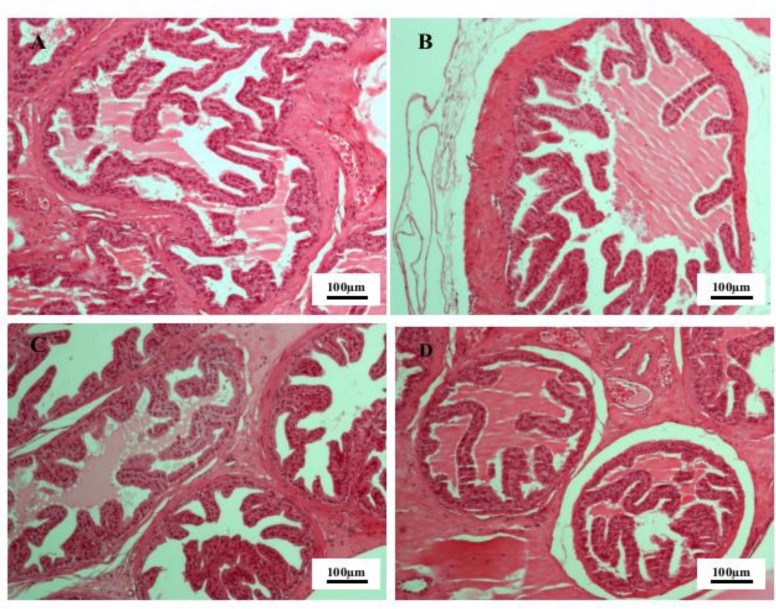
The seminal vesicle tissue in the control group (A) group I (B) group II (C) and group III (D) of male rats. Hematoxylin and eosin staining, 10x magnification

**Figure 4:**
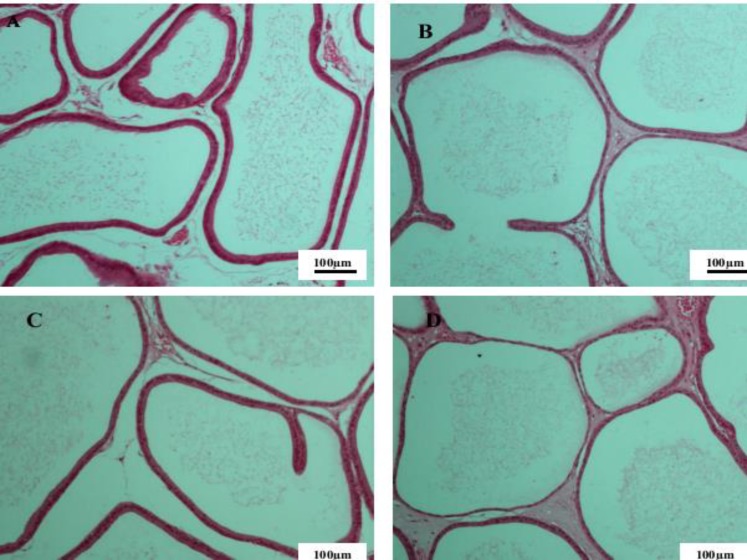
The prostate tissue in the control group (A) group I (B) group II (C) and group III (D) of male rats. Hematoxylin and eosin staining, 10x magnification

Furthermore, no noticeable histopathological change was observed in seminal vesicle and prostate. The tubules in the seminal vesicle showed normal pseudostratified columnar epithelium. In prostate tissue a normal tubuloalveolar structure with normal epithelium and smooth muscles fibers observed in all groups (Figures 3 and 4). 

**Table 3:** The number, weight (g) and crown-rump length (cm) of offspring from mating of control, group I, group II and group III male rats, (n=6 for each group) with 24 female untreated rats

**Groups**	**Number of offspring**	**Weights of offspring**	**Crown Ramp Length of offspring**
**Control**	8.50 ± 1.04 (51)	5.46 ± 0.15	5.80±1.23
**Group I**	9.50 ± 2.42 (59)	6.57 ± 0.23	6.20±1.19
**Group II**	8.33 ± 1.03 (50)	5.56 ± 0.13	6.00±1.23
**Group III**	9.16 ± 1.47 (55)	5.63 ± 0.15	5.90±1.24

Values represent mean ± SEM. There was not statistical difference between the data of different groups.

No statistically significant difference was observed in the weight and CRL of offspring and the pregnancy outcome of control and experimental male rats mated with untreated female rats (Table 3).

## Discussion

Spearmint is a herbal plant which is usually recommended for treatment of many diseases, particularly digestive system problems; however, some studies show that despite its beneficial effects, spearmint has adverse effects on the reproductive system of male rats (Akdogan et al., 2004b ▶; Kumar et al., 2008 ▶). Spearmint reduces free testosterone concentration of serum and has been introduced as an anti-androgenic agent and proposed for treatment of hirsuitism in polycystic ovarian syndrome (PCOS) of women (Akdogan et al., 2007 ▶; Grant, 2010 ▶). Therefore, this study was designed to investigate the effects of spearmint extract on the male reproductive system and fertility outcome.

One of the methods to determine toxicity of plant extracts is measurement of any changes in the body weight (Gupta and Sharma, 2006 ▶). Given no alteration in the body weight of experimental rats in our study, this medicinal plant has no general toxicity effects at the level of administered doses; this circumstance was observed by other studies (Guney et al., 2006 ▶; Kumar et al., 2008 ▶).

For successful fertility, normal structure and accurate function of all parts of reproductive system is needed. A complex mechanism under the regulated function of the hypothalamic-pituitary-gonadal axis (HPG axis) is responsible for initiation and maintenance of spermatogenetic activity. Initially, by secretion of GnRH (gonadotropin-releasing hormone) from the hypothalamus, FSH and LH are released from the pituitary gland. In the testes, under the stimulatory action of LH, the Leydig cells, located in interstitial tissue, produce and secrete testosterone. Simultaneously, FSH supports the function of sertoli cells, a mediator for effects of testosterone and FSH on germ cells for successful spermatogenesis in seminiferous tubules.

 After that, produced sperm pass through the epididymis, which secretes substances for sperm maturation (Cooper, 2002 ▶). Ultimately, through the secretion of substances such as fructose, citrate, inositol and prostaglandins from seminal vesicles and secretion of prostatic liquid from the prostate, semen is produced. Thus any pathological changes in male reproductive tissues may interfere with fertility by altering the level of testosterone hormone or disturbing the spermatogenesis and sperm maturation process (Hafez and Hafez, 2005 ▶). In this study, weight and structure of testes did not change in experimental groups compared to the normal group, which resulted in normal testosterone level and also sperm concentration and motility, two critical parameters for male fertility. As epididymis, seminal vesicle and prostate are androgen-dependent tissues, the normal histology of these tissues as seen in control and experimental groups is the consequence of normal concentration of serum testosterone hormone (Nieschlag et al., 2000 ▶). 

For evaluation of the fertility outcome of male rats, we also determined the number of their offspring born from mating of animals of all 4 groups with untreated females. Since spearmint administration did not cause any decline in number and motility of sperms, no difference was seen in offspring numbers of experimental male rats compared to control. Moreover, the weight and crown-rump length of offspring were not affected. 

Therefore, we concluded that treatment of male rats with the mentioned doses of spearmint extract has no pathological, antiandrogenic or antifertility effects. This result is in contrast with the results of some investigations, which showed that spearmint has an antiandrogenic effect in male rats (Akdogan et al., 2004b ▶; Kumar et al., 2008 ▶). In their studies they used higher doses of spearmint extract and treated animals in a different way. They steeped spearmint tea (dried leaves) in a cup of boiling water and added it to the drinking water. So animals received spearmint continuously at all times for maximum 35 days, while we administered spearmint in lower doses, once a day for a longer time (45 days). Kumar (2008) observed no significant damage to the reproductive system following the **short-term** use of spearmint, but long-term use caused irreversible damage to this system, such as significant decrease in the weights of seminal vesicle, epididymis, testis and prostate with significant histopathological changes in these tissues. Also the level of LH and FSH decreased, which was attributed to generation of oxidative stress in the hypothalamus and pituitary gland, leading to reduced production of GnRH and gonadotropins and resulting in an attenuated level of testosterone and spermatogenesis arrest in treated rats. In addition, severe histopathologic changes were observed in testicular tissue. Similar results were obtained in the study of Akdogan (2004b) ▶ with the same doses and same duration of experiment. But higher levels of LH and FSH were observed in experimental animals followed by decreases in testosterone level and deficiency of spermatogenesis. They explained that an increase in FSH and LH is a normal process which occurs as the result of a decrease in plasma total testosterone levels and concluded that the deficiency of spermatogenesis is the consequence of the direct effect of spearmint on testicular tissue and Leydig cell dysfunction. 

In conclusion, the administration of spearmint at the dosage level used in the present study has no antifertility effect in adult male rats. However, high levels of this extract have adverse effects on male fertility. Therefore, people who consume spearmint should be advised to use this herbal plant in a proper manner and avoid high doses. Furthermore, because the exact constituents of spearmint which cause antifertility have not been clarified, we suggest that ongoing studies should be performed on different fractions of spearmint extract.
